# Topical Application of Hyaluronic Acid-RGD Peptide-Coated Gelatin/Epigallocatechin-3 Gallate (EGCG) Nanoparticles Inhibits Corneal Neovascularization via Inhibition of VEGF Production

**DOI:** 10.3390/pharmaceutics12050404

**Published:** 2020-04-28

**Authors:** Takuya Miyagawa, Zhi-Yu Chen, Che-Yi Chang, Ko-Hua Chen, Yang-Kao Wang, Guei-Sheung Liu, Ching-Li Tseng

**Affiliations:** 1 Graduate Institute of Biomedical Materials and Tissue Engineering, College of Biomedical Engineering, Taipei Medical University, Taipei City 110, Taiwan or michael_idea@hotmail.com (T.M.); or d79010340217@gmail.com (Z.-Y.C.); or orzdinoqoo1@gmail.com (C.-Y.C.); 2 B CUBE, Center for Molecular Bioengineering, Technische Universität Dresden, Tatzberg 41, 01307 Dresden, Germany; 3 Department of Ophthalmology, Taipei Veterans General Hospital, Taipei City 112, Taiwan; Khchen@vghtpe.gov.tw or; 4 Department of Ophthalmology, School of Medicine, College of Medicine, Taipei Medical University, Taipei 11031, Taiwan; 5 Department of Cell Biology and Anatomy, College of Medicine, National Cheng Kung University, Tainan City 701, Taiwan; humwang@ncku.edu.tw; 6 Menzies Institute for Medical Research, University of Tasmania, Hobart, TAS 7000, Australia; 7 Ophthalmology, Department of Surgery, University of Melbourne, East Melbourne, VIC 3002, Australia; 8 International Ph. D. Program in Biomedical Engineering, College of Biomedical Engineering, Taipei Medical University, Taipei City 110, Taiwan; 9 Research Center of Biomedical Device, College of Biomedical Engineering, Taipei Medical University, Taipei city 110, Taiwan; 10 International Ph. D. Program in Cell Therapy and Regenerative Medicine, College of Medicine, Taipei Medical University, Taipei City 110, Taiwan

**Keywords:** anti-angiogenesis, corneal neovascularization (NV), epigallocatechin gallate (EGCG), gelatin, hyaluronic acid (HA), nanoparticles, RGD peptide, eye drops

## Abstract

Neovascularization (NV) of the cornea disrupts vision which leads to blindness. Investigation of antiangiogenic, slow-release and biocompatible approaches for treating corneal NV is of great importance. We designed an eye drop formulation containing gelatin/epigallocatechin-3-gallate (EGCG) nanoparticles (NPs) for targeted therapy in corneal NV. Gelatin-EGCG self-assembled NPs with hyaluronic acid (HA) coating on its surface (named GEH) and hyaluronic acid conjugated with arginine-glycine-aspartic acid (RGD) (GEH-RGD) were synthesized. Human umbilical vein endothelial cells (HUVECs) were used to evaluate the antiangiogenic effect of GEH-RGD NPs in vitro. Moreover, a mouse model of chemical corneal cauterization was employed to evaluate the antiangiogenic effects of GEH-RGD NPs in vivo. GEH-RGD NP treatment significantly reduced endothelial cell tube formation and inhibited metalloproteinase (MMP)-2 and MMP-9 activity in HUVECs in vitro. Topical application of GEH-RGD NPs (once daily for a week) significantly attenuated the formation of pathological vessels in the mouse cornea after chemical cauterization. Reduction in both vascular endothelial growth factor (VEGF) and MMP-9 protein in the GEH-RGD NP-treated cauterized corneas was observed. These results confirm the molecular mechanism of the antiangiogenic effect of GEH-RGD NPs in suppressing pathological corneal NV.

## 1. Introduction

Corneal neovascularization (NV) is the formation of new vessels from pre-existing vascular structures in the transparent cornea, resulting from a variety of ocular pathologic conditions which are detrimental to vision [1,2,3,4]. These newly-formed vessels sprouting from the capillaries of the pericorneal plexus may block light, compromise visual acuity, cause inflammation and corneal scarring, and may eventually result in blindness [2,5,6,7,8]. Etiologies of corneal NV include infection, injury, surgery, autoimmune disease, inflammation, neoplasm, dystrophy, deficiency of limbal barrier function, corneal hypoxia, and improper use of contact lenses [9,10,11,12]. There were over 150 million people worldwide wearing contact lenses in 2019, implying a large population is at risk of developing corneal NV due to corneal hypoxia [13,14,15,16,17,18]. To combat this, vascular endothelial growth factor (VEGF) targeting antigen-binding fragment has been developed to treat NV; it demonstrates great promise for the treatment of corneal NV [2]. New drug/compounds for inhibiting vessel formation have also been developed to effectively treat NV-related pathological corneal conditions [19].

Green tea is one of the most popular beverages in the world. Early studies have indicated that the consumption of green tea can inhibit inflammation and angiogenesis [19]. The major active component of green tea is a catechin-derived polyphenol, including (–)-epigallocatechin gallate (EGCG), which has been shown to inhibit angiogenesis via inhibition of vascular endothelial cell growth [19,20,21,22,23,24,25]. The EGCG has also been shown to effectively limit the upregulation of metalloproteinase (MMP)-9 and VEGF in a mouse model of corneal NV treated by subconjunctival injection of EGCG [23]. Therefore, EGCG was chosen to treat corneal NV in this study. During vascular remodeling and formation in damaged and regenerated tissues, several integrins are found to be expressed in vascular endothelial cells [26,27,28,29]. Among them, αvβ3 integrins are involved in ocular angiogenesis [27]. The adhesion molecule integrins, αvβ3, plays an important role in angiogenesis, and several studies have shown that arginine-glycine-aspartic acid (RGD) peptides particularly recognize αvβ3 integrins on the tumoral endothelial cell membrane and newly-formed blood vessels during angiogenesis [27,28,29,30,31]. In a previous study, we reported that RGD peptide modified nanoparticles (NPs) can specifically deliver EGCG to human umbilical vein endothelial cells (HUVEC) [20]. Therefore, RGD-based targeting strategy could be used to enhance biomaterial-endothelial cell interaction [29,30,32] to target pathological angiogenesis.

Conventional methods of ocular drug delivery include topical administration, intravitreal injection, and intraocular implant [17,33,34,35]. The topical application with an eye drop formulation represents a common, noninvasive approach for ocular drug delivery. The major drawbacks of eye drops include poor ocular drug bioavailability, nasolacrimal duct drainage, and poor penetration to the posterior segments of the eye [17,34,35]. Therefore, seeking a next-generation delivery material/strategy becomes an urgent issue in the context of eye drop delivery. Recently, biodegradable NPs have been applied to ophthalmic research for less invasive and cheaper intervention alternatives [3,34,35,36]. The application of NPs on ocular diseases allows targeted delivery, slow-release, and enhanced pharmacokinetics, thereby improving the bioavailability of drugs in the eyes [35,36]. However, the detailed therapeutic mechanism and performance for this nanomedicine for corneal NV have not yet been elucidated.

**Figure 1:**
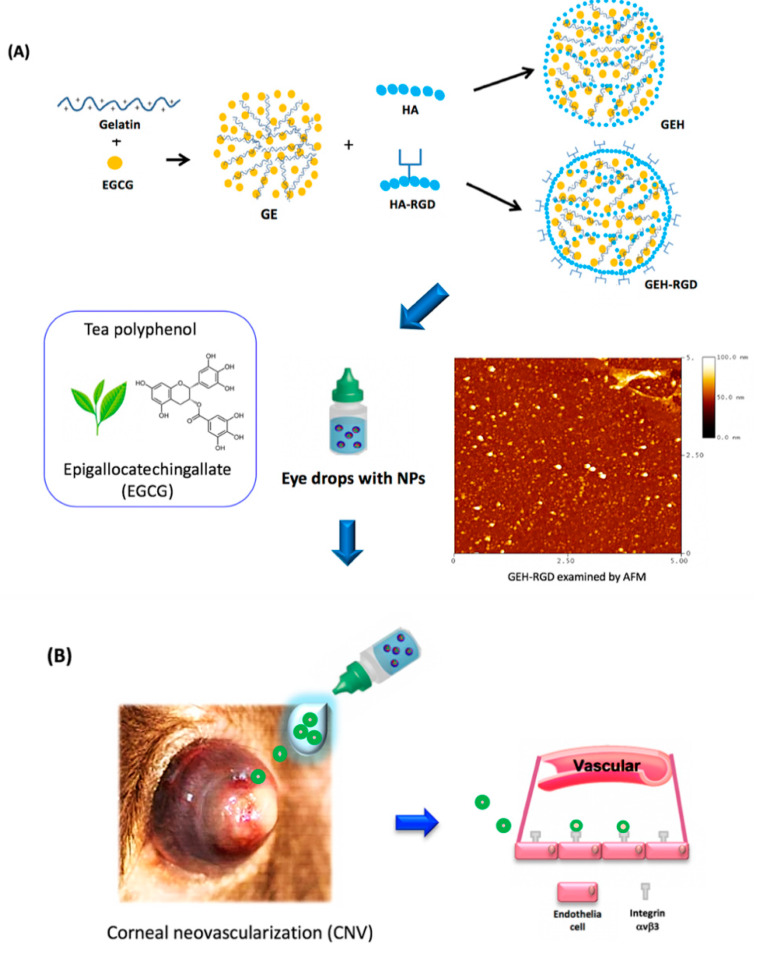
Schematic illustration of this study. (**A**) Synthesis of GEH-RGD NPs with EGCG loading and (**B**) application in corneal NV treatment as an eye drop formulation. Abbreviation: EGCG: epigallocatechin-3 gallate, HA: hyaluronic acid, NPs: nanoparticles, RGD: arginine-glycine-aspartic acid, GE: Gelatin/EGCG self-assembling NPs, GEH: GE NPs with HA surface coating, GEH-RGD: GE NPs with HA-RGD surface decoration, AFM: atomic force microscope.

In this study, we applied RGD-modified NPs containing EGCG as an eye drop formula for the treatment of corneal NV. We conducted an in vitro functional assay using HUVECs as our cellular model system. In addition to in vitro study, we also employed a mouse model of chemical cauterization-induced corneal NV to investigate the antiangiogenic effect of RGD-modified NPs and their underlying molecular mechanisms in corneal NV inhibition in vivo. This topical nanomedicine could be a potential therapeutic alternative for the treatment of corneal NV (a schematic of this study is shown in Figure 1).

## 2. Materials and Methods

### 2.1. Reagents and Chemicals

HUVECs were purchased from Bioresource Collection and Research Center (Hsinchu, Taiwan) and grown in Medium 199 containing 10% fetal bovine serum (Thermo Fisher Scientific, Waltham, MA, USA) as well as Penicillin-Streptomycin (Life technologies, Eugene, OR, USA) and endothelial cell growth supplement (ECGS) (Merck Millipore, Darmstadt, Germany) at 37 °C under 5% CO_2_ in a humidified incubator. Upon reaching 90% confluence, cells were trypsinized with 0.25% (*w/v*) trypsin/1 mM EDTA (Gibco BRL, Gaithersburg, MD, USA) and split for further use. Gelatin type A (bloom 110, from porcine skin), EGCG (≥95%), 1-Ethyl-3-(3-dimethyllaminopropyl) carbodiimide hydrochloride (EDC), and N-Hydroxysuccinimide (NHS) were purchased from Sigma-Aldrich (St. Louis, MO, USA). One percent highly purified sodium hyaluronate (defined molecular weight 600~1200 kDa) in 3 mL was obtained from Maxigen (ArtiAid®, Maxigen Co. Ltd., Wu-gu district, New Taipei City, Taiwan). H-Gly-Arg-Asp-Ser-Pro-Lys-OH (GRGDSPK) was acquired from MDBio, Inc. (Shandong, China). Succinimidyl ester (TAMRA-SE) mixed isomers (a fluorescence dye) and 5(6)Carboxytetramethyl rhodamine were acquired from Thermo Fisher Scientific (Waltham, MA, USA). The topical anesthesia solution (0.5% Alcaine®) was from Alcon-Couvreur (Puurs, Belgium). Grafco® Silver Nitrate Applicators were purchased from Medline Industries Inc. (Mundelein, IL, USA). All other reagent-grade chemicals were from Sigma-Aldrich.

### 2.2. Preparation of Gelatin/EGCG NPs with Surface HA-RGD-Conjugation (GEH-RGD)

The HA solution (5 mg/mL, 2mL) was added to the EDC solution (38 mg/mL, 1mL) and mixed at room temperature (RT) for 1 h. Then, 1µL of GRGDSPK peptide in 0.1 M NaHCO_3_ (10 mg/mL) solution was added for peptide-HA conjugation [20]. This reaction was kept at 4 °C for 72 h. Nonreacting residues were removed by centrifugation, the purified solution finally was lyophilized, and the dried power of HA-RGD conjugation was obtained. The identification of conjugation by ^1^H-nuclear magnetic resonance (NMR) and Fourier-transform infrared spectroscopy (FTIR) was described in a previous study [20]. An equal volume of gelatin and EGCG solution (both in 0.44 *w/v* %) was mixed gently to form the self-assembly NPs under stirring, named GE hereafter [20,37]. Surface-modified NPs were then prepared, and 100 µL of HA or HA-RGD was separately added into the GE NP suspension (final HA concentration, 0.25 *w/v* %). GE with HA coating on the surface is referred to as GEH hereafter, and GEH-RGD is the abbreviation for GE with HA-RGD peptide modifications on the surface. A schematic representation of the preparation process is shown in Figure 1A. The synthesized NPs were then characterized by dynamic light scattering (DLS) for particle size and zeta potential measurement. Similar to our previous study [20], the ζ-potential of GE is positive (+18 mV). After applying the HA coating (GEH), the ζ-potential of GEH became negative (−13 mV) due to the HA possessing carboxyl groups (–COO^−^). When HA-RGD was added to the particle surface, a positive ζ-potential of GEH-RGD (+12.9 mV) was acquired, since the side chain of GRGDSPK peptide present many amide (–NH_3_^+^) groups on HA-RGD. This is one way to confirm the RGD on the particle surface. The encapsulation efficiency of EGCG was determined by reacting with cation-radicals of 2,2′-azino-bis (3-ethylbenz -othiazoline-6-sulfonic acid) diammonium salt (ABTS) (ABTS+, Sigma-Aldrich, St Louis, MO, USA; Supplemental-1 in Appendix A) [37,38]. The EGCG loading rate in GEH or GEH-RHD NPs was around 95%. The EGCG loaded NPs prepared from three batches (*n* = 6) were used in this test. The morphology of nanoparticles was examined by an MFP-3D atomic force microscope (AFM, Asylum Research, Santa Barbara, CA, USA) using tapping-mode. GEH-RGD NPs with EGCG and free-form EGCG were freshly prepared for the experiments.

### 2.3. Functional Evaluation of GEH-RGD NPs on HUVECs

#### 2.3.1. Tube Formation Assay

HUVECs were treated with EGCG, GEH, and GEH-RGD NP solution (EGCG: 20 µg/mL), and then seeded on a Matrigel®-coated 96-well plate (Corning, Corning, NY, USA). The morphology of tube formation was observed and images in each treatment were taken at 9 and 24 h (*n* = 3). Images were acquired using an inverted fluorescence microscope (Olympus, IX81, Tokyo, Japan). The branch points and tubule length were quantified by ImageJ (http://imagej.nih.gov/ij/; provided in the public domain by the National Institutes of Health, Bethesda, MD, USA).

#### 2.3.2. Gelatin Zymography

HUVECs were treated with EGCG, GEH, and GEH-RGD (20 µg/mL) containing media for 24 h, and then the media was harvested. Preparation of separating gel included gelatin type A solution, (20 mg/mL, 1% *w/v* SDS), followed by sample loading and gel running. After gel electrophoresis, separating gel was incubated in 2.5% Triton X-100-containing incubation buffer for 20 h in an incubator at 37 °C. The gel was then stained 0.05% Coomassie Brilliant Blue G-250 for an hour. After gel destaining, the gel was photographed, and the gelatinolytic area of each image was quantified by ImageJ (*n* = 3).

### 2.4. Topical Delivery of NPs in a Mouse Model of Corneal NV

C57BL/6J male mice aged from 8 to 10 weeks were used in this study. The experimental procedure was performed following the ARVO Statement for the Use of Animals in Ophthalmic and Vision Research and approved by the Institutional Animal Care and Use Committee (IACUC) of the Taipei Medical University (IACUC approval no. LAC-10-0289, 9 May 2013). Briefly, mice were anesthetized, followed by pressing the tip of an applicator containing silver nitrate to the center of the cornea to generate chemical cauterization. Each mouse only suffered one eye cauterization. The nanoparticles containing eye drops (GEH or GEH-RGD NPs) were diluted in PBS to adjust the EGCG concentration to 30 µg/mL for use in this test (Figure 1B). The free drug (EGCG solution) was also prepared in the same EGCG concentration. Five microliters of tested eye drops were applied to the eye of the mice once a day for seven days, and PBS topical application was used as vehicle control (*n* = 6/group). The burn response and the severity of NV were assessed by a hand-held portable slit lamp (SL-17, Kowa Company Ltd., Torrance, CA, USA) on anesthetized mice. Four batches of the animal experiment were performed in this study. Seven days after cauterization, the extent of NV was assessed in the anaesthetized mice by an ophthalmologist masked to the treatments under a slit-lamp and dissecting microscope. The cauterized cornea was observed under a hand-hold portable slit lamp and each quadrant was photographed. The image of the cauterized cornea was then processed using ImageJ software (http://imagej.nih.gov/ij/) to quantify the NV area through the following steps: 1) remove the eyeball background and retain the corneal area; 2) use the RGB function to remove a nonred color area (nonvessel area); 3) calculate the vascular area (in red) among the total corneal area and present it as a percentage. The area of corneal NV was calculated by averaging the four quadrants of the cornea.

#### 2.4.1. Histopathology Examination of Corneal Sections

Mouse eyes were harvested and fixed in 10% formaldehyde. The extracted eyeballs were embedded in paraffin and cut into 5-µm-thick sections, deparaffinized and hydrated, and stained with hematoxylin and eosin (H&E stain). The sections were reviewed and evaluated under a light microscope.

#### 2.4.2. Quantification of VEGF and MMP-9 in Cornea Extraction

Corneal tissues (6 eyeballs/group from two batches) were harvested and homogenized with protein extraction buffer (Thermo Fisher Scientific). The mixture from each sample was then centrifuged, and the supernatant was collected. Total protein of cornea lysate was quantified by Bradford assay (p010, GeneCopoeia, Rockville, MD, USA). An equal amount of total protein (15 µg in 100 µL) from each sample was used to quantify the VEGF and MMP-9 by ELISA (Quantikine ELISA kits, R&D system, Minneapolis, MN, USA). This experiment was conducted according to the manufacturer’s protocol.

### 2.5. Statistical Analysis

All data are shown as mean ± standard deviation (SD) from three independent experiments. Statistical differences between groups were tested by Student’s t-test or one-way ANOVA using SPSS 17.0 (SPSS, Inc., Chicago, IL, USA). A probability (p) value less than 0.05 was considered statistically significant.

## 3. Results

### 3.1. Characterization of EGCG-Loaded GEH-RGD NPs

The gelatin/EGCG NPs (GE) were first formed by self-assembling. The surface of NPs was then decorated with HA or HA-RGD, termed GEH and GEH-RGD, respectively (Figure 1A). The particle size of GE, GEH, and GEH-RGD was 91.90 ± 44.53, 277.40 ± 73.00, and 158.10 ± 11.06 nm, respectively (Table 1). The GE presented a positive surface with a zeta (ζ) potential value at 18.4 ± 4.4 mV. The ζ potential of GEH and GEH-RGD were the opposite. GEH was −13.2 ± 4.1 mV, and GEH-RGD was 12.9 ± 4.1 mV. All NPs with low PDI value presented as monodispersed colloidal systems with narrow size distribution. An image of GEH-RGD acquired from AFM examination (in Figure 1A) revealed round particle deposition on the surface with no aggregation and proper dispersion.

### 3.2. GEH-RGD NPs Inhibit In Vitro Angiogenetic Activity

**Figure 2:**
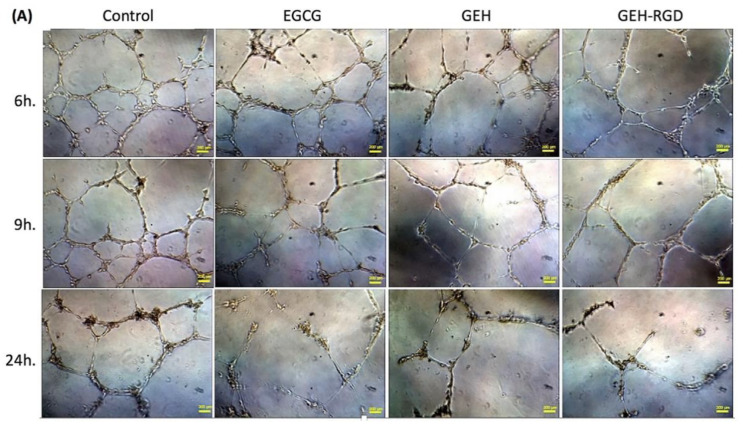

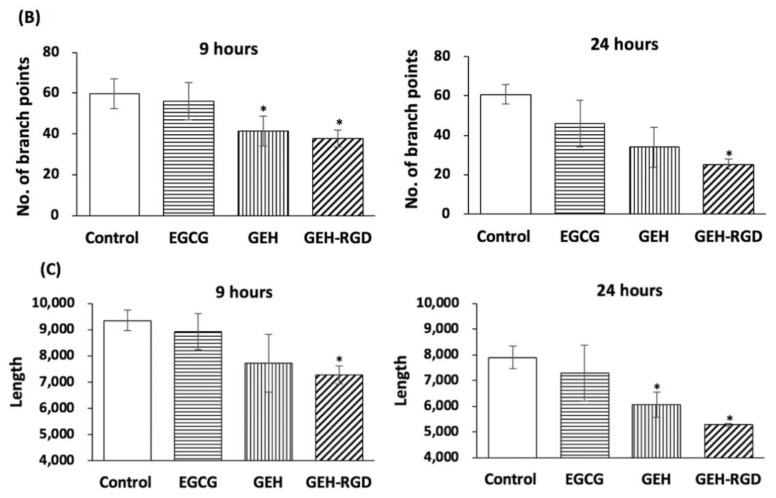
EGCG NPs inhibit endothelial tube formation. (**A**) Representative images of HUVECs cultured on Matrigel at a different time point (EGCG: 20 µg/mL, 100×). The images in each treatment were taken at 9 and 24 h, and (**B**) the number of branch points and (**C**) total length of tubing cells were quantified (*n* = 3). *p < 0.05 compared with control. Abbreviation: PBS: Phosphate buffer saline, EGCG: epigallocatechin-3 gallate, HA: hyaluronic acid, RGD: arginine-glycine-aspartic acid, GE: Gelatin/EGCG self-assembling.

The in vitro tube formation assay was performed to evaluate the effect of EGCG NPs on angiogenesis. When HUVECs were cultured on Matrigel, they gradually formed capillary-like tubular structures which connected to each other, arranging themselves in a mesh-like network (Figure 2A, control). The network gradually disappeared with time and less capillary structure was observed when cocultured with variant EGCG formula addition (Figure 2A, EGCG/GEH/GEH-RGD). Almost no mesh-like structure was found in the GEH-RGD NP-treated cells (Figure 2A). The GEH-RGD-treated group has a smaller number of branch points (25.3 ± 2.5, Figure 2B) and the shortest tubule length (5284.3 ± 54.6, Figure 2C) compared with the control group (60.7 ± 4.9; 7896.7 ± 437.6) at 24-hour time points (*p < 0.05) (Figure 2B,C). Together, these results demonstrate that GEH-RGD NPs can effectively inhibit angiogenic activity in vitro.

### 3.3. GEH-RGD NPs Inhibit MMP-2 and MMP-9 Activities

**Figure 3:**
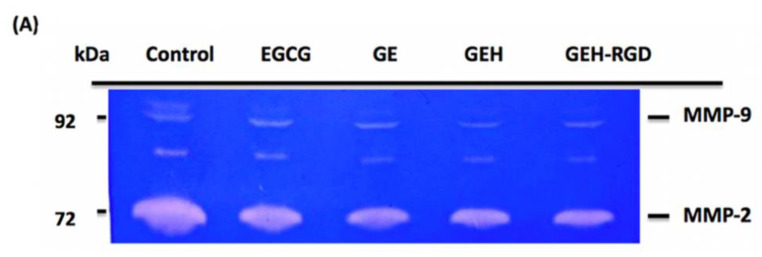

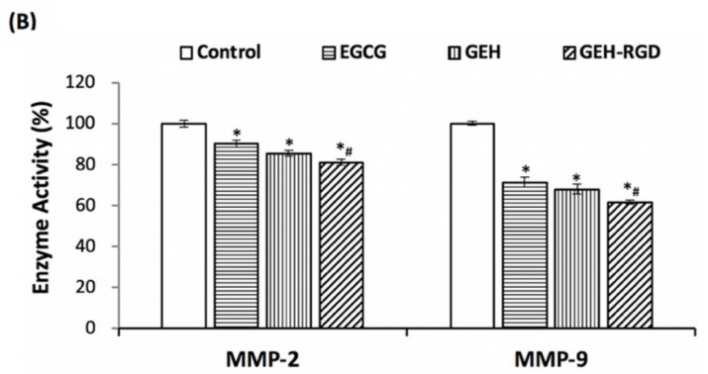
EGCG NPs inhibit activity of MMPs in endothelial cells. (**A**) Results of gelatin zymography from culture medium after 24 h incubation with a variant formulation for confirm the MMPs activity. (**B**) Quantification of MMP-2 and MMP-9 activities compared with the control group (*n* = 3). *p < 0.05 compared with control, ^#^*p* < 0.05 compared with EGCG. Abbreviation: EGCG: epigallocatechin-3 gallate, HA: hyaluronic acid, RGD: arginine-glycine-aspartic acid, GE: Gelatin/EGCG self-assembling nanoparticles (NPs), GEH: GE NPs with HA surface coating, GEH-RGD: GE NPs with HA-RGD surface decoration, MMP: metalloproteinase.

The formation of corneal NV is closely associated with the activity of MMPs, such as MMP-2 and MMP-9. We examined the effect of EGCG NPs on the activity of MMPs secreted from the treated HUVECs via gelatin zymography (Figure 3A,B). The gelatinolytic of the control group (only culture medium) was normalized as 100%. Cells treated by EGCG revealed the gelatinolytic activity of MMP-2 at 90.1 ± 1.6% and MMP-9 at 71.4 ± 2.4%. Conditioned media harvested from GEH-RGD-treated cells had a lower gelatinolytic activity of both MMP-2 (81.1 ± 1.5%) and MMP-9 (61.1 ± 1%) compared to cells treated with other groups (*p < 0.05 for control, #*p* < 0.05 for EGCG). Our results indicate that GEH-RGD NPs inhibit the activity of MMPs, which contributes to the inhibition of angiogenesis.

### 3.4. Topical Application of GEH-RGD NPs Suppresses the Corneal NV in a Mouse Model of Chemical Cauterization

**Figure 4:**
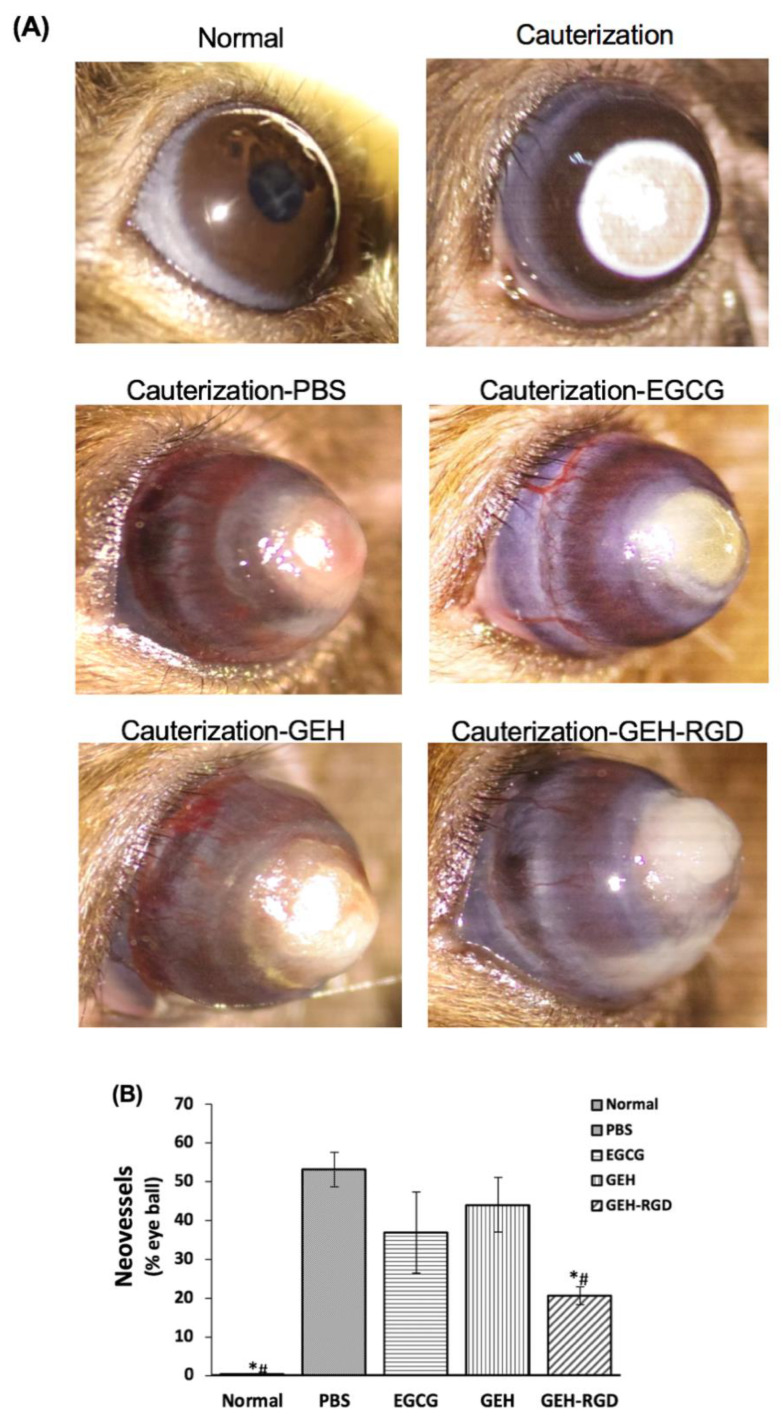
EGCG NPs inhibit neovessels formation in chemical cauterization-induced corneal neovascularization (NV). (**A**) Representative images of normal cornea, cauterized cornea and PBS-, EGCG-, GEH-, or GEH-RGD-treated cornea on day 7 following chemical cauterization. (**B**) The area of blood vessels in the cornea was quantified (*n* = 6). *p < 0.05 compared with PBS, ^#^*p* < 0.05 compared with EGCG and GEH. Abbreviation: PBS: Phosphate buffer saline, EGCG: epigallocatechin-3 gallate, HA: hyaluronic acid, RGD: arginine-glycine-aspartic acid, GE: Gelatin/EGCG self-assembling nanoparticles (NPs), GEH: GE NPs with HA surface coating, GEH-RGD: GE NPs with HA-RGD surface decoration.

To further investigate whether the above in vitro findings were applicable in vivo, we then tested the antiangiogenic effect of EGCG, GEH and GEH-RGD NPs in a mouse model of chemical cauterization-induced corneal NV. The normal cornea was in a transparent and smooth surface, as seen in Figure 4A. A white or cloudy patch on the center of the cornea accompanied by swelling was observed immediately after chemical cauterization (Figure 4A, cauterization). Chemical cauterization results in a growth of new blood vessels from limbus toward the burn scar. Dense ingrown vessels surrounding the entire eyeball were observed from the corneal limbus to the burning edge in the PBS (Figure 4A) and EGCG-treated groups acquired on day 7 (Figure 4A, EGCG). In contrast, fewer and thinner visible NVs were observed in both GEH and GEH-RGD-treated groups (Figure 4A). Moreover, better corneal transparency with the least amount of vessel formation was found in the GEH-RGD-treated group compared to other treatment groups.

The quantification of NV areas in the cornea is shown in Figure 4B. The pathological blood vessels absent in healthy cornea tissue was normalized as 0%. Our results indicate a good therapeutic potential of GEH-RGD NPs, which shows the lowest NV area (20.7 ± 2.2%. *p < 0.05) compared with PBS (53.2 ± 4.5%), while the EGCG- and GEH-treated group had a higher NV area (37.0 ± 10.5% and 40.1 ± 7.1%, ^#^*p* < 0.05) (Figure 4G).

**Figure 5:**
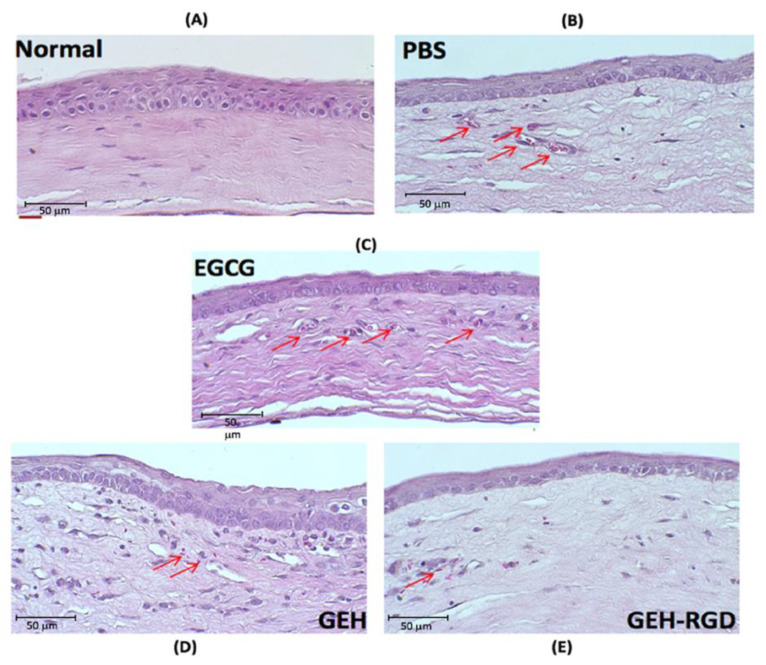
Histological assessment of corneal sections after treatment. Representative images of the central corneal section were depicted on day 7 following chemical cauterization. The development of fibrovascular proliferation was observed (red arrows). Groups: (**A**) normal, (**B**) PBS, (**C**) EGCG, (**D**) GEH, (**E**) GEH-RGD. Abbreviation: PBS: Phosphate buffer saline, EGCG: epigallocatechin-3 gallate, HA: hyaluronic acid, RGD: arginine-glycine-aspartic acid, GE: Gelatin/EGCG self-assembling nanoparticles (NPs), GEH: GE NPs with HA surface coating, GEH-RGD: GE NPs with HA-RGD surface decoration.

We then evaluated the microstructure of corneas after treatment with GEH-RGD NPs. The outer part of normal mouse cornea is composed of 3–5 layers of epithelium cells, bowman’s membrane, and stroma (Figure 5A). After chemical cauterization, a thinner corneal epithelium was found in the PBS, EGCG, GEH and GEH-RGD-treated groups (Figure 5B–E). We observed relatively loose and irregular structures of stroma and more newly-formed blood vessels in the PBS, EGCG, and GEH-treated groups (Figure 5B–D). In contrast, the GEH-RGD-treated group showed a relatively normal stroma and reduced newly-formed vessel formation (Figure 5E). Therefore, these results demonstrate that GEH-RGD NPs can effectively prevent the development of NV in the cornea after chemical cauterization.

### 3.5. Topical Application of GEH-RGD NPs Attenuates the Expression of VEGF and MMP-9 in the Chemical Cauterized Corneas

**Figure 6:**
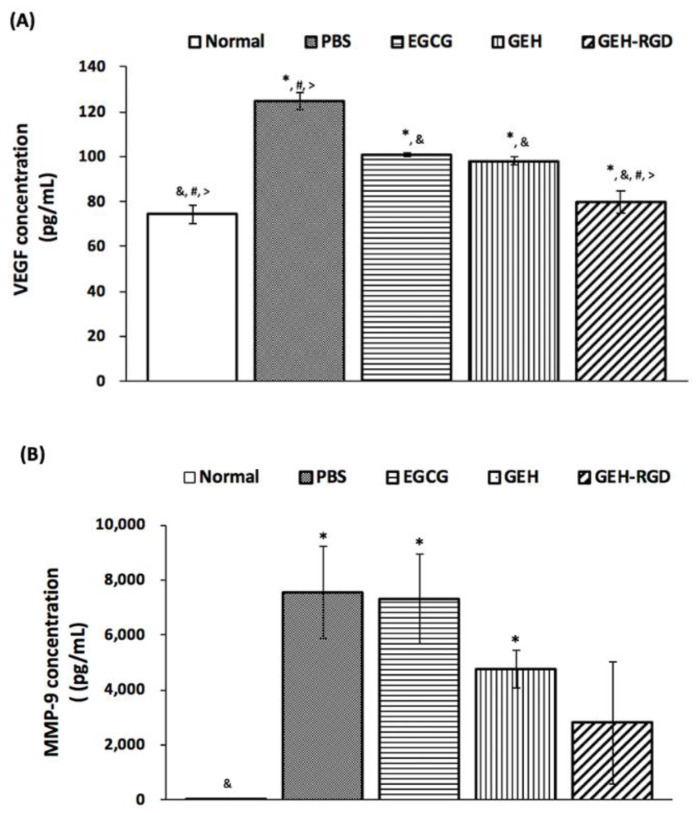
GEH-RGD NPs inhibit the expression of angiogenic factors in the cauterized cornea. Mice received chemical cauterization and were treated with PBS, EGCG, GEH NPs, GEH-RGD NPs-contained eye drops once daily for 7 days. The corneas were harvested and homogenized, and the protein level of (**A**) VEGF or (**B**) MMP-9 were assayed by ELISA (6 eyeballs/group from two batches). (*p < 0.05 compared with control, ^&^*p* < 0.05 compared with PBS, ^#^*p* < 0.05 compared with EGCG, ^>^*p* < 0.05 compared with GEH). Abbreviation: PBS: Phosphate buffer saline, EGCG: epigallocatechin-3 gallate, HA: hyaluronic acid, RGD: arginine-glycine-aspartic acid, GE: Gelatin/EGCG self-assembling nanoparticles (NPs), GEH: GE NPs with HA surface coating, GEH-RGD: GE NPs with HA-RGD surface decoration. MMP: metalloproteinase, VEGF: vascularization endothelium growth factor, ELISA: enzyme-linked immunosorbent assay.

To further elucidate the factors that contribute to the GEH-RGD NP-mediated angiogenesis inhibition in the cauterized corneas, the protein level of VEGF and MMP-9 protein in the cauterized corneas were measured by ELISA. Normal corneas showed a concentration of (74.3 ± 4.0 pg/mL) for VEGF, while the amount of MMP-9 was almost undetectable in corneal tissues. The PBS-treated corneas showed the highest concentration of VEGF (124.6 ± 3.8 pg/mL) and MMP-9 (7554 ± 1678 pg/mL) compared to all other treated groups after chemical cauterization (Figure 6A,B). The VEGF level was significantly reduced in the EGCG- (100.8 ± 0.8 pg/mL), GEH- (98.1 ± 1.8 pg/mL) and GEH-RGD-treated cauterized corneas (79.9 ± 5.0 pg/mL). The VEGF protein in the cauterized corneas with GEH-RGD NPs treatment was reduced to the level similar to normal corneas (*p < 0.05 compared with control, ^&^*p* < 0.05 compared with PBS, ^#^*p* < 0.05 compared with EGCG, ^>^*p* < 0.05 compared with GEH; Figure 6A). Moreover, the MMP-9 level in the cauterized cornea treated with GEH NPs and GEH-RGD NPs were 4762 ± 680 pg/mL and 2800 ± 2326 pg/mL, respectively. In contrast, EGCG solution had no effect still representing high MMP-9 concentration (7295 ± 1630 pg/mL), similar to the PBS-treated corneas (Figure 6B). These results indicate that GEH-RGD NPs inhibit corneal NV by inhibiting the production of VEGF and MMP-9 in chemical-cauterized corneal tissue.

## 4. Discussion

The importance of this study lies in the demonstration that RGD-HA conjugation on the gelatin/EGCG NP surface to target integrin can decrease the angiogenic activity in human endothelial cells. Our data also suggest that the GEH-RGD NP eye drop formulation is superior to EGCG free drug and nontargetable GEH NPs, as it can effectively inhibit corneal NV in a mouse model of chemical injury.

EGCG is a dual-functional agent which has both antiangiogenic and anti-inflammatory capacity [25,37,38,39,40,41]. EGCG has also been shown to inhibit angiogenesis by regulating endothelial cell growth, thereby reducing pathological corneal NV [18]. Green tea extract inhibits the angiogenesis of human endothelial cells through the reduction of expression of VEGFR [42]. Sánchez-Huerta et al. revealed that the administration of EGCG to the ocular surface can suppress corneal NV due to its ability to mediate a variety of inflammatory and angiogenic factors such as interleukin-1β, cyclooxygenase 2 (COX2), VEGF, and MMPs [43]. Our previous study (Chang C.Y et al. 2017, [20]) and current data suggest that EGCG, GEH, and GEH-RGD NPs can suppress the angiogenesis activity of HUVECs, especially the GEH-RGD NPs.

Many eye diseases can be treated by eye drops. The major disadvantages of eye drop dosage include tear screening, nasolacrimal duct drainage, and corneal tight junction as a barrier that reduces drug bioavailability in the eyes [33]. The application of a nanoformulation for ocular drug delivery allows targeted transportation and slow drug release, as well as enhancing drug retention in the eye, thereby improving the bioavailability of drugs. Positively-charged nanoparticles with a diameter of 250 nm consisting of EGCG with surface decoration by HA resulted in increased tear volume, reduced inflammatory gene expression, and the restoration of a normal corneal architecture with improving associated clinical signs [37]. The HA-RGD conjugated gelatin/EGCG NPs were around 160 nm in size, and the ζ-potential presented a positive value at 12.9 mV (Table 1), which was in a similar range to what was observed in our previous study, indicating that the synthetic quality for producing GEH-RGD NPs is stable [20].

Under normal conditions, VEGF promotes endothelial migration and proliferation, which helps to maintain normal vasculatures by preventing the apoptosis of endothelial cells [43]. Our results have also demonstrated that EGCG NPs inhibit endothelial cell migration (Supplemental-2 in Appendix A). However, overexpression of VEGF is associated with several vascular eye diseases such as diabetic retinopathy [44], corneal NV [2,3], and choroidal NV [45]. For endothelial targeting, cell surface markers such as P-selectin, E-selectin, vascular cell adhesion molecule-1, and integrin are considered potential target moieties [46,47,48,49]. One of integrin with subunit in αvβ3 type is important in mediating angiogenesis, blocking αvβ3 function which reduces the blood flow to certain tumors [29,30,31]. Moreover, normal epidermis and corneal epithelium lack expression of α5β1 and αvβ3 integrins [27]. Therefore, targeting RGD would not misrecognize the integrin expression on vascular endothelial cells on the cornea. According to the specific targeting capacity of RGD [20], our designed GEH-RGD NPs can be specifically uptaken by human endothelial cells and modulate the angiogenic activities with a long-lasting effect due to the slow release of EGCG from GEH-RGD [20].

The antiangiogenic effect of GEH-RGD and its underlying molecular mechanism was confirmed by employing a chemical cauterized mouse model. Chemical injury is a prevalent cause of corneal NV clinically due to easy vessel observation [50]. In this study, corneal NV was induced by silver nitrate cauterization to obtain a robust in vivo model for mimicking the clinical condition of corneal injury. Considering that topical application is the most accessible and least invasive delivery route to the ocular surface, a GEH-RGD NPs eye drop formulation was designed and manufactured. In this study, EGCG concentration for the treatment of corneal NV was 30 μg/mL, given once a day for 7 days. Due to the slow release of EGCG from GEH-RGD NPs, only one dose per day can achieve the therapeutic effect, i.e., inhibiting the formation of new blood vessels. The GEH/GEH-RGD NPs were synthesized from biocompatible materials, i.e., gelatin and hyaluronic acid; these materials possess good biocompatibility and prolonged ocular retention time [20,51].

Angiogenesis requires MMPs to dissolve the basement membrane to initial endothelial sprouting. EGCG was found to decrease VEGF receptor phosphorylation and inhibit the secretion of MMP-2 and MMP-9 in human endothelial cells [52]. Lee, H.S. et al. reported that EGCG could be an inhibitor of ocular angiogenesis. They reported that EGCG can attenuate the expression of pro-angiogenic factors (such as MMP-9 and VEGF) by inhibiting the generation of reactive oxygen species in human retinal pigment epithelial cells, and block angiogenic activity in human retinal microvascular endothelial cells [23]. In this study, our results show that GEH-RGD NPs blocked the activity of MMP-2 and MMP-9 in HUVECs in vitro and reduced the MMP-9 and VEGF proteins in chemical cauterized corneas in vivo. Interestingly, we did not observe a significant difference in MMP-9 activity between GEH and GEH-RGD NPs. Since MMP-9 is secreted by a range of cell types, including immune cells and fibroblasts, GEH NPs can interact with corneal cells and block the MMP-9 activity of the surrounding cells by releasing EGCG. This noncell type-specific effect of EGCG may also be found in cornea treated with GEH-RGD NPs. Overall, these data indicate that the mechanism underlying the inhibitory effects of GEH-RGD NPs was, at least, through the reduction of MMPs and VEGF expression, and that further antiangiogenesis effect in the damaged cornea was achieved.

Koh CH et al. evaluated the topical delivery of 0.1% EGCG eye drops 4 times daily for 2 weeks in a rabbit model of silk suture-stimulated corneal NV [53]. They reported that topical administration of EGCG effectively inhibits corneal vessel formation in rabbits via suppression of VEGF and COX-2 [53]. In our study, a chemical cauterized mouse model was used and the targetable nanoformulation, GEH-RGD, as eye drops can effectively inhibit corneal NV with one dosage per day at a very lower concentration (30 μg/mL, 0.03% *w/v*). One study using curcumin-loaded polyethylene glycol-block-polycaprolactone (PEG-PCL) NPs to treat corneal NV in mice with alkaline burned reveals that this treatment can also successfully decrease vessel formation in the cornea using nano-curcumin once daily up to 2 weeks [54]. These results suggest that using these specific nanoparticle-encapsulated drug releasing systems as eye drops can reduce the dosing frequency thanks to the advantages of nanoparticle interaction with the ocular surface to achieve higher drug bioavailability to effectively inhibit the formation of vessels.

## 5. Conclusions

In conclusion, our study confirms the antiangiogenic effects of GEH-RGD NPs in vitro by inhibiting vascular endothelial cells function. Our data evidence that an eye drop formulation with GEH-RGD NPs can effectively target corneal vessels and thereby inhibit chemical cauterized-induced corneal NV by a once-daily treatment. These findings suggest that the topical application of GEH-RGD NPs is a potential therapeutic approach for the management of corneal NV.

## Figures and Tables

**Table 1:** Characterization of variant NPs.

NP’s Group	Particle Size (nm)	Zeta Potential (mV)	PDI
GE	91.90 ± 44.53	18.4 ± 4.4	0.30 ± 0.20
GEH	277.40 ± 73.00	−13.2 ± 4.1	0.38 ± 0.18
GEH-RGD	158.10 ± 11.06	12.9 ± 4.1	± 0.03

Values represent: mean ± standard division (*n* = 6); PDI: Poly dispersive index; EGCG: epigallocatechin-3-gallate; NPs: nanoparticles; RGD: arginine-glycine-aspartic acid; GE: Gelatin/EGCG self-assembling NPs; GEH: GE NPs with hyaluronic acid (HA) surface coating; GEH-RGD: GE NPs with HA-RGD surface decoration.

**Figure A1:**
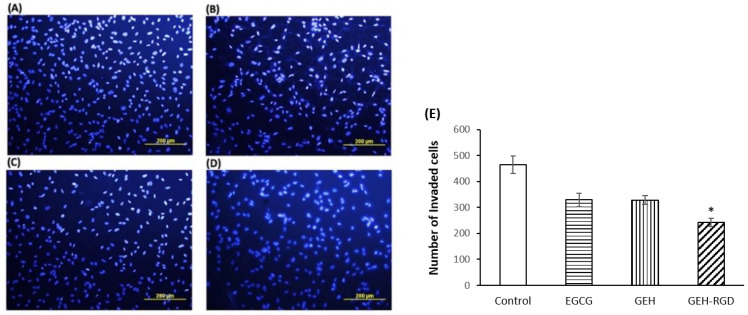
EGCG NPs inhibit endothelial cell migration. Representative images of HUVECs invaded across the membrane treated by (**A**) culture medium as control, (**B**) EGCG solution, (**C**) GEH NPs, and (**D**) GEH-RGD NPs at EGCG concentration of 20 µg/mL (*n* = 3). (**E**) Numbers of invaded cells were calculated using ImageJ. *p < 0.05 compared with control, ^#^*p* < 0.05 compared with GEH. Abbreviation: EGCG: epigallocatechin-3 gallate, HA: hyaluronic acid, RGD: arginine-glycine-aspartic acid, GE: Gelatin/EGCG self-assembling nanoparticles (NPs), GEH: GE NPs with HA surface coating, GEH-RGD: GE NPs with HA-RGD surface decoration, HUVECs: human umbilical vein endothelial cells.

## Appendix A

### Appendix A.1. Supplement 1—The Protocol for EGCG Quantification by ABST+ Method

The supernatant with EGCG after centrifugation was assayed by determining unloaded EGCG content with ABTS+ method. Added 88 μL K_2_S_2_O_8_ solution (0.14 M) into 5 mL of ABTS stock solution (7 × 10^−3^ M), this mixture was kept overnight at room temperature for working solution (ABTS+) preparation. Two microliter EGCG contained sample was added into the 800 μL ABTS+ working solution reacted for 24 h., then measured the absorbance at 734 nm by Varioskan Flash spectral scanning multimode reader (Thermo Fisher Scientific, Waltham, MA, USA). The OD value of sample was compared with the EGCG standard curve to calculate the concentration [38]. The EGCG encapsulation efficiency (EE) was counted by calculated as the following equation: EE % = (Total amount of EGCG − Free EGCG)/(Total amount of EGCG) × 100.

### Appendix A.2. Supplement 2—Transwell Migration Assay

HUVECs (1.2 × 10^4^ cells/well) were seeded in the Transwell® (Corning, Corning, NY, USA) then putting in a 24-well plate. In the upper chamber, HUVEC cells were incubated with EGCG, GEH NPs, and GEH-RGD NPs (EGCG: 20 μg/mL) overnight. After migration, cells on top of the transwell membrane were removed and the remaining cells pass through the transwell membrane were fixed with 10% formaldehyde. Nuclei were stained with 4’,6-diamidino-2-phenylindole (DAPI, Thermo Fisher Scientific, Waltham, MA, USA). Images were acquired using an inverted fluorescence microscope (Olympus, IX81, Tokyo, Japan) and the number of migrated cells was quantified using ImageJ (*n* = 3). Here, it is suggested that GEH-RGD NPs significantly inhibit endothelial migration through the transwell membrane, less cells nuclei were observed (Figure A1A–D) and counted (Figure A1E, **p* < 0.05 compared with control).
